# Cellulose-coated emulsion micro-particles self-assemble with yeasts for cellulose bio-conversion

**DOI:** 10.1038/s41598-024-56204-0

**Published:** 2024-03-06

**Authors:** Ester Korkus Hamal, Gilad Alfassi, Margarita Antonenko, Dmitry M. Rein, Yachin Cohen

**Affiliations:** 1 Department of Chemical Engineering, Technion - Israel Institute of Technology, 3200003 Haifa, Israel; 2 Department of Biotechnology Engineering, Braude College of Engineering, Karmiel, Israel

**Keywords:** Biotechnology, Environmental sciences, Engineering

## Abstract

In the quest for alternative renewable energy sources, a new self-assembled hybrid configuration of cellulose-coated oil-in-water emulsion particles with yeast was formed. In this research, the addition of yeasts (*S. cerevisiae*) to the micro-particle emulsion revealed a novel self-assembly configuration in which the yeast cell is connected to surrounding cellulose-coated micro-particles. This hybrid configuration may enhance the simultaneous saccharification and fermentation process by substrate channeling. Glucose produced by hydrolysis of the cellulose shells coating the micro-particles, catalyzed by cellulytic enzymes attached to their coating, is directly fermented to ethanol by the yeasts to which the particles are connected. The results indicate ethanol yield of 62%, based on the cellulose content of the emulsion, achieved by the yeast/micro-particle hybrids. The functionality of this hybrid configuration is expected to serve as a micro-reactor for a cascade of biochemical reactions in a “one-pot” consolidated process transforming cellulose to valuable chemicals, such as biodiesel.

## Introduction

Ethanol (bioethanol) made from lignocellulosic biomass holds great potential for reductions of environmental pollution compared to fossil fuels. Current methods of producing bioethanol are mainly by fermentation of glucose obtained from enzymatic hydrolysis of cellulose. Over the years, there has been significant development to improve the enzymatic hydrolysis of cellulosic materials^1,2^. Enhanced enzymatic hydrolysis yield can be achieved by suitable pre-treatment, such as using aqueous alkali^3^ or low cost protic ionic liquids (PIL)^4^. Moreover, a previous study has shown that the enzymatic hydrolysis rate of cellulose-coated emulsion particles, fabricated by homogenization of hydrocarbons with a suspension of regenerated cellulose hydrogel particles, exhibits similar to that of the cellulose hydrogel suspension, both being significantly enhanced compared to hydrolysis of micro-crystalline cellulose. The rate of enzymatic hydrolysis of the cellulose shell encapsulating the emulsion particles was sensitive to the cellulose/oil ratio of the particles' composition^5^.

Simultaneous saccharification and fermentation (SSF) has been proposed as a productive approach to improve enzymatic hydrolysis and to enhance ethanol yield^6^. In separate hydrolysis and fermentation processes (SHF) each reaction can take place at optimum conditions for the enzyme and the yeast. However, the enzyme is inhibited when the products, glucose and cellobiose accumulate. On the other hand, in the SSF process there is demand to compromise the optimum conditions for hydrolysis and fermentation simultaneously^6–9^. Furthermore, the effect of both ethanol and yeast on cellulase activity and hydrolysis of crystalline cellulose was investigated in the last decade^10^. Ethanol in the concentration range between 1 and 7% inhibits the enzymatic hydrolysis of cellulosic materials. However, the yeast has no direct influence on the enzymatic activity^11^. Wang et al. have shown that the combination of novel pretreatment method with engineered yeast can significantly improve ethanol yields from cellulosic biomass feedstocks^12,13^.

Emulsified SSF process with cellulose-coated emulsion micro-particles, at various suitable conditions (a variety of yeasts and temperatures) has recently been investigated as a method for rapid and efficient ethanol production. This emulsified SSF process achieved high ethanol yields (close to the theoretical cellulosic ethanol yields) by increasing the saccharification rate at temperatures tolerable to the yeast (30 °C)^14^. There is currently a significant effort in metabolic engineering of yeasts to achieve multi-enzyme transformation of cellulose to bio-fuel^15–18^. Alternatively, hybridization of the yeasts with cellulosic substrate incorporated with cellulytic enzymes, may achieve this effect without metabolic engineering. Due to the significant importance of the various implementations of yeast fermentation, several groups studied methods to improve production of ethanol by yeast fermentation such as, immobilization of yeast cells^19–33^. A study on the effect of using immobilized *S. cerevisiae* on bacterial cellulose (BC) for ethanol production showed that BC may be a suitable substrate for yeast immobilization due to its unique properties: high adsorption capacity, porosity, and large surface area^30,34^. Liu et al. have engineered *S. cerevisiae* with four different heterologous expressions of cellulases for application in the saccharification and fermentation process for biofuel production. They achieved a direct strategy to produce ethanol from rice straw by increasing the interactions of the engineered yeast cells with cellulose^28^.

Herein, we establish and examine a unique process, hybridized simultaneous saccharification and fermentation (hSSF), in which the yeasts are hybridized by self-assembly with emulsion micro-particle encapsulated by a cellulose shell. In this manner, the yeast-micro-particle hybrid provides substrate channeling in a multi-enzyme consolidated bioprocess^35^. We foresee that integrating the micro-particles of this hybrid system with cellulase, and lipase, the yeast-micro-particle hybrid can further function as a micro-bioreactor for a "one-pot" process transforming cellulose to valuable chemicals, such as biodiesel.

## Results

### Self-assembly of yeast cells and cellulose-coated o/w emulsion particles

**Figure 1:**
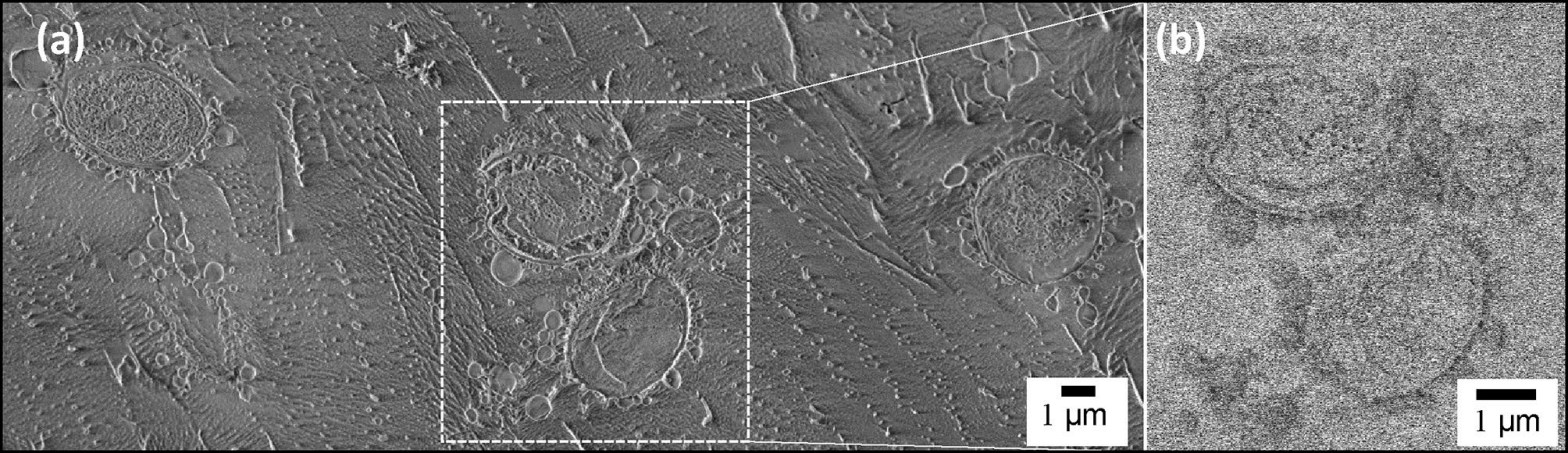
Cryo-SEM images of the fractured surface of a vitrified emulsion droplet containing cellulose-coated micro-particles integrated with 1 wt.% dispersed *S. cerevisiae*. The emulsion was fabricated by HPH at 10,000 psi, cellulose:castor oil wt. ratio 1:1. Imaging performed using: (**a**) InLens detector (**b**) ESB detector. The cryo-fracture plane passes through the yeast cell.

**Figure 2:**
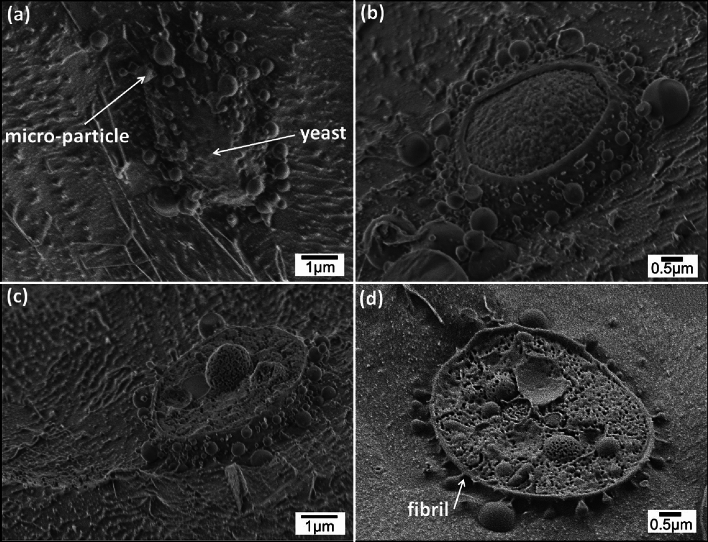
Cryo-SEM images of the fractured surface of a vitrified emulsion droplet containing cellulose-coated micro-particles integrated with 1 wt.% dispersed *S. cerevisiae*. The emulsion was fabricated by HPH at 10,000 psi. The *S. cerevisiae* cell is surrounded by micro-particles made at cellulose:castor oil wt. ratio: 1:1 (**a**–**c**) and 1:6 (**d**). The cryo-fracture plane passes above the top of the yeast cell in (**a**), just below it in (**b**) and through the yeast cell in (**c**, **d**).

The unique self-assembly of yeasts (*S. cerevisiae*) with oil in water (o/w) micro-particles, emulsified by encapsulation with unmodified cellulose, was examined using castor oil as the hydrophobic phase and coated at cellulose:oil weight ratios of 1:1 and 1:6. Cryo-SEM imaging of the fractured surface of specimens was used to characterize the structure of the hybrid system of cellulose-coated castor oil emulsion particles integrated with 1 wt.% yeast dispersion. Cryo-SEM images, after some sublimation of the surrounding frozen water, are exhibited in Figs. 1 and 2. The images reveal emulsion particles with a circular shape with clear visual borders and yeast cells which are surrounded by the cellulose-coated emulsion particles. Imaging contrast using ESB (energy-selective back scattered electrons) detector, based on the elemental composition of the specimen, allows us to distinguish between the emulsion particles (darker areas) and yeast cell (brighter areas) (Fig. 1b). Direct interaction between the cellulose-coated emulsion particles and the yeast cell is observed in Figs. 1 and 2. This adhesion seems to occur by formation of fibrils connecting the yeast cell wall with the cellulose shell of the emulsion particles (Fig. 2d). The external shell of the cellulose-coated emulsion particles contains cellulose hydrogel while the yeast cell wall includes polysaccharides β-glucans and chitin among other components^36–39^. The adhesion between the emulsion particles and yeast cell, as shown in Figs. 1 and 2, may be explained by direct interaction between the cellulose hydrogel and the components of the yeast cell wall. Figure 2a exhibits an intact portion of an entrapped yeast cell with cellulose-coated emulsion particles. In other cases, such as shown in Fig. 2b, the yeast cell wall was removed during fracture of the frozen samples. The inner structure of the yeast cell is clearly observed in Fig. 2c and d. The particles’ diameter ranges from 0.1 to 1 µm, while the yeast cell dimensions range from 3 to 6 µm. The particle dimensions evaluated by cryo-SEM are in accord with those observed in light and fluorescence microscope images (Figures S1 and S2, respectively). Moreover, light scattering measurements performed on hybridized system of cellulose-coated o/w emulsion with castor oil in the core with 1 wt.% yeast dispersion as well as the yeast dispersion alone, (Figure S3) exhibit good correlation with the cryo-SEM images.

#### Hybridized simultaneous saccharification and fermentation

**Figure 3:**
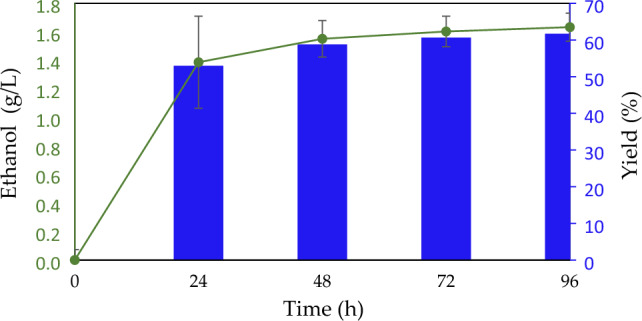
Ethanol concentration and yield during hSSF of emulsions containing cellulose-coated micro-particles with incorporated enzymes, hybridized with dispersed *S. cerevisiae*.

This innovative system of yeast cells hybridized with cellulose-coated o/w emulsion particles fulfills a micro-environment for an effective hSSF process. It combines enzymatic hydrolysis of the aqueous cellulose hydrogel shell followed by fermentation of the released glucose in close proximity with yeast cells on micron-scale dimensions. The hSSF process to produce ethanol was monitored throughout 96 h and analyzed using GC. Figure 3 demonstrates ethanol production of 1.6 g/L. Calculation of the ethanol yield after 96 h of hSSF process provide 62% of the theoretical yield based on the weight of cellulose in the emulsion particles. The ethanol yield achieved compares favorably with other reports^16^.

## Discussion

Integration of cellulose-coated o/w emulsion with yeast cell expose a unique self-assembly configuration: hybrids of yeast cells connected by fibrils to surrounding micro-particles. Cryo-SEM imaging provides evidence of this configuration. It exhibits direct interaction of emulsion particles and yeast cell. In this research, we demonstrate, surprisingly, that the cellulose-coated emulsion-yeast cell adhesion occurs spontaneously in the emulsion-yeast system, whereas most previous works have reported on various treatments for immobilization of yeast in cellulose^40–42^. It was found that immobilization of yeast in a cellulose matrix improved the cellulose adsorption capacity for separation application^43–45^. Moreover, this system is stable and active for an effective SSF process. The adhesion between the components of this system may enhance the SSF process by minimizing ethanol dilution into the surrounding aqueous medium, while removing the hydrolysis product from the active enzymes. However, a direct effect of the emulsion particle and yeast structure on the enzyme activity was not observed, in accord with a previous work^11^. This study is a proof-of-concept of the hSSF process and its viability as an effective bioreactor. It is the first work (to our knowledge) that reports on the direct interaction between a cellulose shell of cellulose-coated emulsion particles and the yeast cell wall, by fibrils that connect between them. Further studies are also needed on the formation of the fibrillary connection between the yeasts’ cell wall and the micro-particles’ cellulose shell, its biological origin and structure, and optimization of its function in substrate channeling.

**Figure 4:**
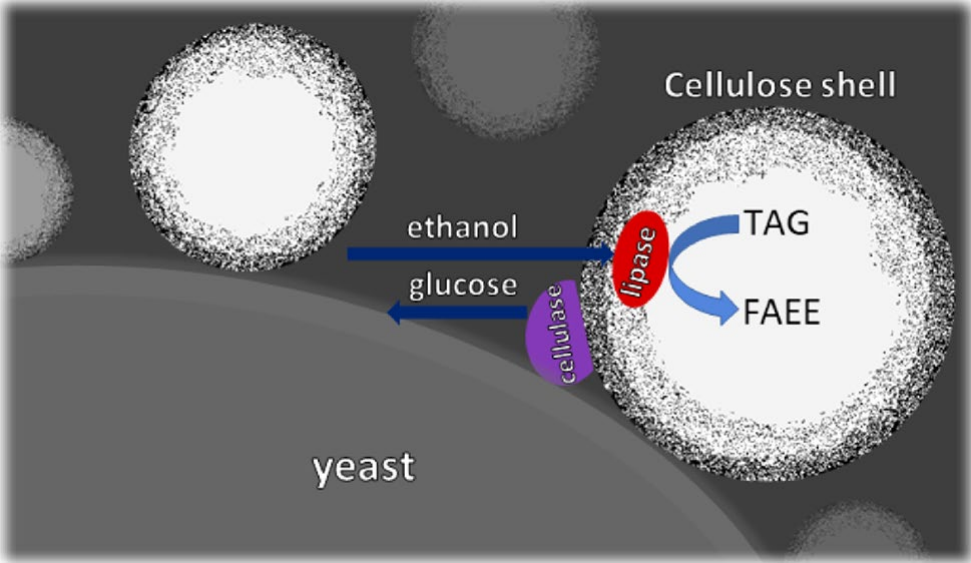
Scheme of cellulose-coated micro-particles with incorporated enzymes, hybridized with yeast, effecting a cascade of biochemical reactions.

Implementation of an aqueous-organic two-phase system may be an enhanced solution for the impediments of cellulosic biofuel production. In particular, for significantly improved product separation. A scheme of the one-pot process of transformation of cellulose to fatty-acid ethanol ester (FAEE, biodiesel) using cellulose-coated o/w emulsion particles as bio-reactors is shown in Fig. 4. It presents an effective cascade of biochemical processes of cellulose hydrolysis, glucose fermentation and ethanol-triglyceride trans-esterification, by hybridization of micro-organisms with cellulose-coated micro-particles incorporated with enzymes (cellulytic enzymes and lipase). The unique fibril-mediated adhesion of micro-particles to the micro-organism cell wall may enhance the fermentation and transesterification processes: glucose production at the emulsion particle surface by enzymatic cellulose hydrolysis, fermented to ethanol by the hybridized yeast. The released ethanol should be captured by the surrounding emulsion particles for subsequent transesterification with oil at their core, catalyzed by lipase, assembled at the inner interface between the core oil and the regenerated cellulose shell, to form FAEE. Despite the hybridization, a possible limitation of the hSSF process is that the ethanol yield may be decreased by loss of glucose to the surrounding aqueous medium due to incomplete substrate channeling or un-hybridized micro-particles or yeasts. These issues will be optimized in future studies.

A possible extension of this work is envisioned in conjunction with our previous study on transesterification of castor oil in the core of these emulsion micro-particles with ethanol dissolved in the aqueous medium, catalyzed by lipase within the particles. In this case, the FAEE remained dissolved in the core of the emulsion particles^46^. The self-assembly of cellulose-coated emulsion particles around yeast cells offers close contact on micron-scale dimensions between all components, which is expected to facilitate mass transfer of products and reactants with negligible loss to the surrounding medium. This configuration offers a promising design of an enhanced cascade of environmentally friendly biochemical processes for transformation of cellulose to biodiesel.

## Experimental

### Materials

Micro-crystalline cellulose powder with a particle size in the range 20–160 µm (degree of polymerization ~295 as given by the supplier) was obtained from Sigma Aldrich (Rehovot, Israel). Sodium hydroxide, 1-butanol, yeast (*Saccharomyces cerevisiae*) and β-Glucosidase from *Aspergillus niger* were purchased from Sigma Aldrich (Rehovot, Israel). Castor oil was obtained from Chen Samuel chemicals (Haifa, Israel). Celluclast 1.5 L was Purchased from Novozymes A/S (Bagsvaerd, Denmark).

## Methods

### Emulsion preparation and hybridization with yeast

Molecularly dissolved cellulose solutions (4 wt.%) were obtained by mixing micro-crystalline cellulose in aqueous NaOH (7 wt.%) at room temperature and then in a cooling bath (− 16 °C) using a mechanical stirrer at 500 rpm, until no crystalline cellulose was observed visually. Hydrogel dispersion was achieved by regeneration from its precursor solution by adding deionized water, until electrical conductivity measurements indicated removal of alkali traces in the hydrogel dispersion (below 1 mS cm^-1^).

Emulsions of cellulose-coated castor oil (Castor oil served as a model oil due to its benefits, as it is non-toxic, miscible in alcohol, renewable, biodegradable and in particular being a non-edible oil^46^ micro-particles, containing 1 wt.% cellulose, were obtained by two stages. First, a pre-emulsion was prepared by mixing cellulose hydrogel dispersion (~ 2.5 wt.%), castor oil (at the specified cellulose: oil ratios) and water, using a mechanical homogenizer IKA® T-18 Ultra-Turrax® (IKA Works Inc., USA) at 20,000 rpm for 5 min. The coarse emulsion was subjected to high-pressure homogenization (HPH) using a microfluidizer Model LM-20 (Microfluidics, Westwood MA, USA) at homogenization pressure of 10,000 psi for 4 min. During HPH the temperature was kept around $40^\circ{\rm C}$ by using ice. For ensuing hybridization with the emulsion micro-particles by self-assembly, yeasts *S. cerevisiae)* were added to 10 ml emulsion samples, to achieve a 1 wt.% concentration.

### Imaging by cryogenic scanning electron microscopy (cryo-SEM)

The morphology of cellulose-coated o/w emulsion particles was investigated using a Zeiss Ultra Plus (Carl Zeiss, Jena, Germany) high-resolution cryogenic scanning electron microscopy (cryo-HRSEM) with a Schottky field-emission gun. The cryo-HRSEM is equipped with Bal-Tec VCT100 (Balzers, Liechtenstein) cold stage maintained at temperatures below − 145 °C. The cryo-specimen preparation included placing an emulsion droplet on a stub which was vitrified by plunging into supercooled liquid ethane and then into liquid nitrogen. Then the frozen sample was transferred to a BAF060 freeze fracture unit (Leica Microsystems, Wetzlar, Germany) via a pumped cryo-transfer shuttle, maintained at − 170 °C. A rapidly cooled knife was used to fracture the frozen sample. The fractured sample was transferred to the HRSEM for imaging. Temperature was raised to − 100 °C for 30 s to remove some of the ice by sublimation to expose structural features and improve contrast^47^. The samples were imaged at low electron acceleration voltage (1–1.4 kV) and working distance (3–4.5 mm) to minimize charging. The Everhart–Thornley detector (SE2) and in-the-column (InLens) for high-resolution surface information was used. In addition, elemental contrast was observed in the energy-selective backscattered (“EsB”) detector. The images were examined with imageJ software, scientific image analysis software (U. S. National Institutes of Health, Bethesda, Maryland, USA).

### Hybridized simultaneous saccharification and fermentation (hSSF)

hSSF was performed on hybridized emulsions of 1:6 cellulose:oil wt. ratio Celluclast (30 FPU g^-1^ cellulose), β-glucosidase (30 CBU g^-1^ cellulose) and 5 ml of sodium acetate buffer solution at a pH of 4.8 (50 mmol L^-1^) were added to 15 ml falcon tubes containing 5 ml hybridized emulsion sample. The SSF was carried in a shaker incubator at 40 °C, 120 rpm for 96 h. Samples for gas chromatography (GC) analysis were collected after centrifugation of 5 min at 6000 rpm. All samples were tested in four duplicates.

### Reaction analysis by gas chromatography (GC)

Ethanol concentrations in the reacted SSF samples were determined using GC analysis. It was based on the standard method for quantitative determination of ethanol, but the internal standard that was used in this study is just butanol^48^. The samples were centrifuged for 5 min at 6000 rpm, 6 ml from the clear solution and 100 µl of butanol were transferred into a calibration bottle. Finally, 600 µl of the sample were transferred into a glass vial and injected into a CP-3800 Gas Chromatograph (Varian Inc. Walnut Creek, CA, USA) equipped with a CP-8907 column and a flame ionization detector. Flow rate of 1.1 ml min^-1^ was used. Calculation of the theoretical ethanol yield was done using Equation 2 proposed by Hoffman et al.^14^.

### Supplementary Information

Supplementary Figures.

## Data Availability

All data generated or analyzed during this study are included in this published article and its supplementary information file.
