# Supplementary material for: Cellulose-coated emulsion micro-particles self-assemble with yeasts for cellulose bio-conversion
Source: Sci Rep. 2024 Mar 6;14:5499. doi: 10.1038/s41598-024-56204-0 (PMC10918086; doi:10.1038/s41598-024-56204-0)
Supplement: Supplementary file 1 — Supplementary Figures. [file 41598_2024_56204_MOESM1_ESM.pdf]

Supplementary information

Self-Assembly of Cellulose-Coated Oil in Water Emulsions on Yeast: Novel implementation of bio-reactor for Cellulose to Biodiesel

Ester Korkus Hamal[1*], *Gilad Alfassi*[2], *Margarita Antonenko*[1], *Dmitry M. Rein*[1], *Yachin Cohen*[1]

[1]Department of Chemical Engineering, Technion - Israel Institute of Technology, Haifa 3200003, Israel

[2] Department of Biotechnology Engineering, ORT Braude College, Karmiel, Israel.

* Corresponding author. E-mail address: sesterko@campus.technion.ac.il

Light Microscopy

Light microscope imaging was made with Olympus BH2 light microscope (Olympus, Tokyo, Japan), connected to a 12-bit cooled CCD camera, using Achromat positive low phase contrast objectives. The images were analyzed with imageJ software, scientific image analysis software.

Fluorescence Microscopy

The Fluorescence images were received by the Spinning Disk Confocal microscope from Nikon with CSU-W1 Confocal Scanner Unit. Fluorescence imaging of the yeast surrounded by cellulose-coated emulsions were made by dissolving CFW and NR in water and oil, respectively. With red emission in the hydrophobic environment and blue fluorescence in the cellulose shell. NR is a hydrophobic dye with an excitation a 515-560 nm and emission at wavelengths larger than 590 nm. CFW with excitation at 350 nm and emission at 450-500 nm.

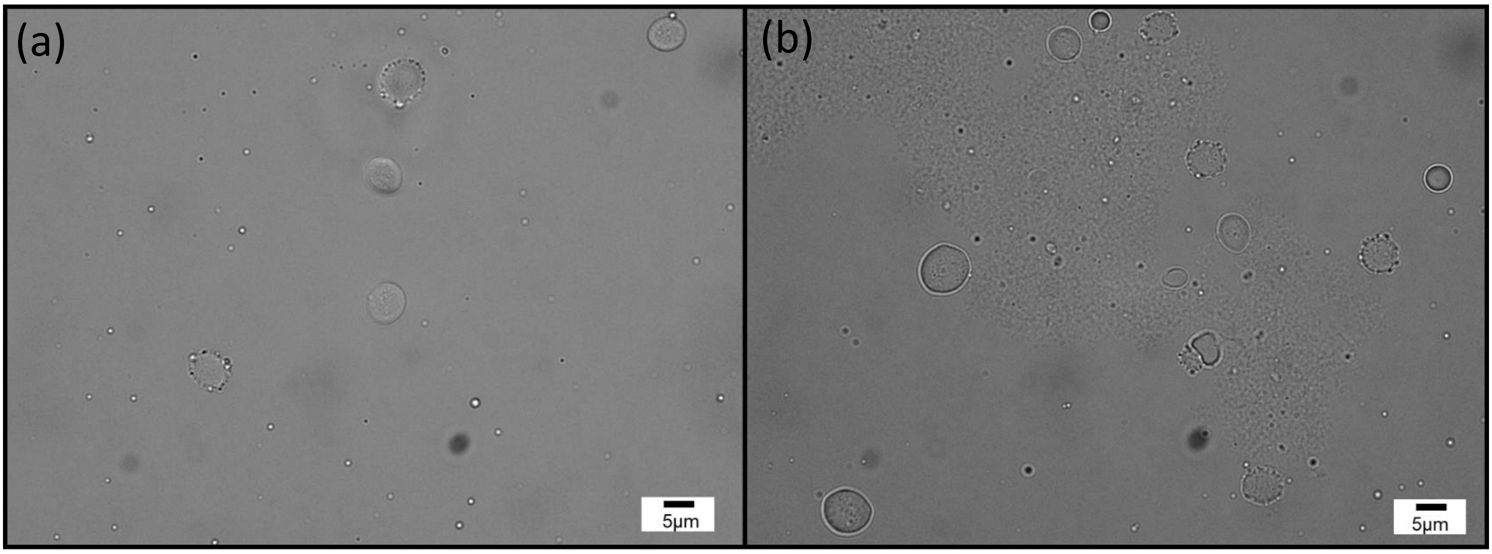

Figure S1. Light microscope images of emulsion containing cellulose-coated micro-particles and 1%wt. dispersed yeast. The micro-particles were made at cellulose:castor oil wt. ratio 1:1 by high-pressure homogenization at 10,000 psi.

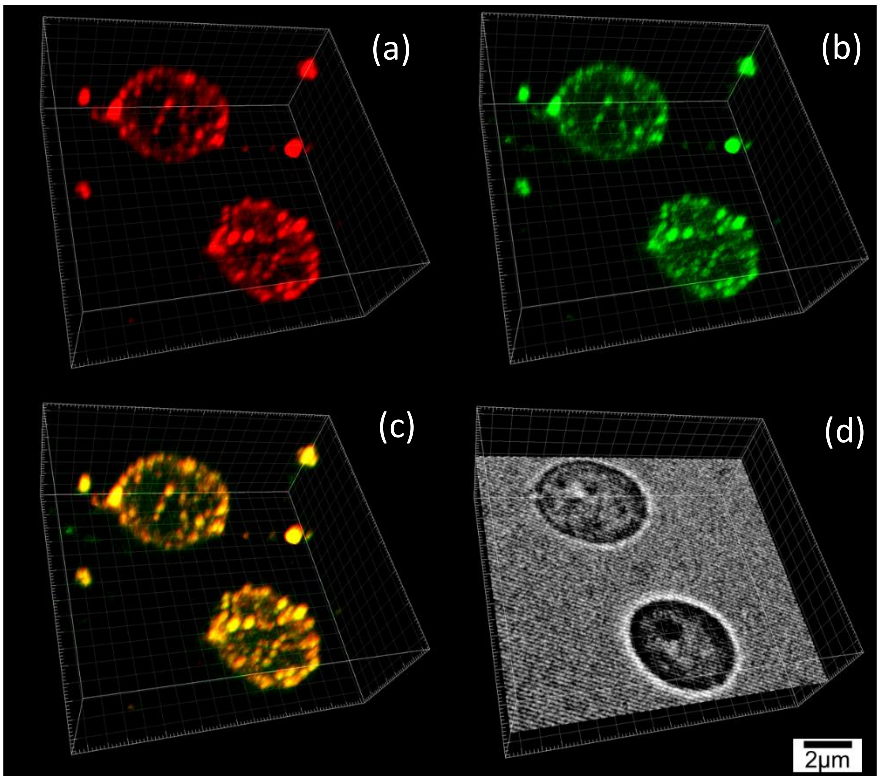

Figure S2. Fluorescence microscope images of the same emulsion as in Figure S1, stained with: a) Nile Red (oil phase); b) Calcofluor-white (shell of cellulose); c) both; d. Phase-contrast image.

Light scattering (LS)

Particle size and size distribution of the cellulose-coated o/w emulsion particles were monitored by light scattering using a Mastersizer 2000 (Malvern Co. Ltd., UK), equipped with He-Ne red laser (λ=633 nm). The particle size distribution was calculated on a volume-average basis. The measurement was performed in triplicate.

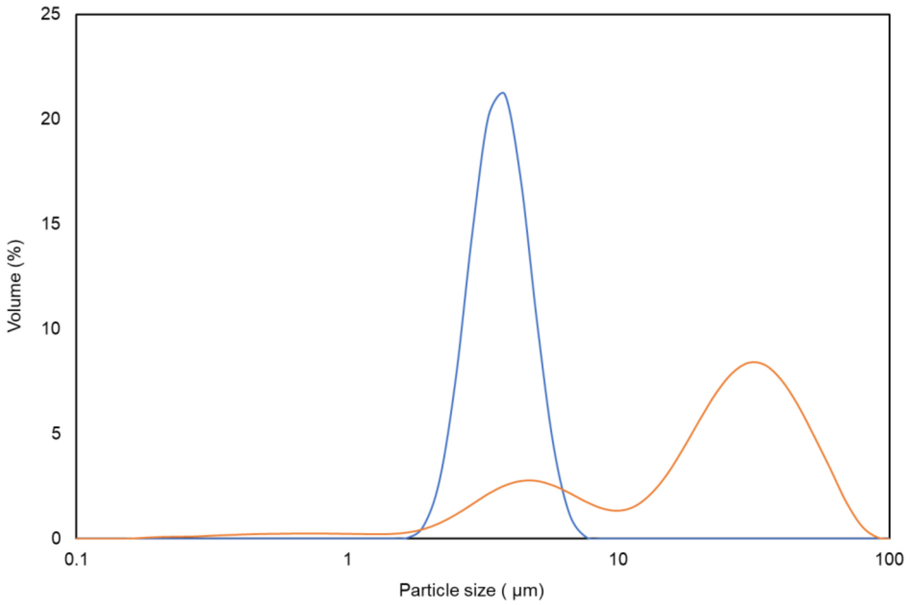

Figure S3. Particle size distributions (by volume) evaluated by light scattering. Blue line: 1%wt. dispersed yeast; Orange line: emulsion containing cellulose-coated micro-particles (cellulose:castor oil wt. ratio 1:1) and 1%wt. dispersed yeast.

GC chromatograms:

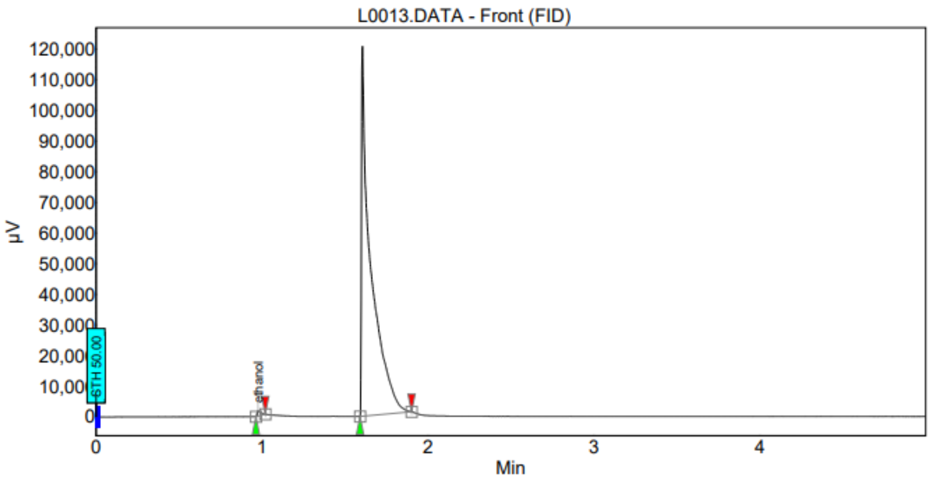

Peak results :

Index	Name	Time [Min]	Quantity [%]	Area [μV.Sec]	#baseline
1	ethanol	0.98	0.00	2402.4	0
2	UNKNOWN	1.61	0.00	469472.0	1
Total			0.00	471874.4	

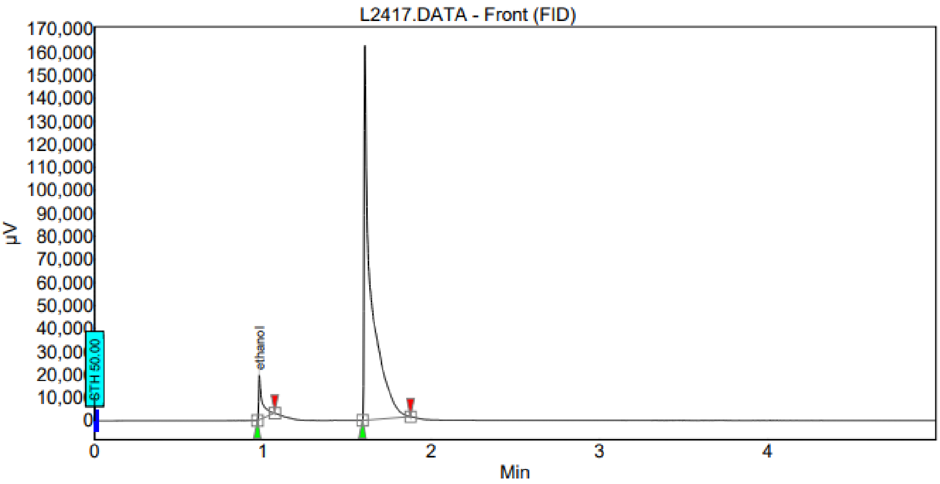

Peak results :

Index	Name	Time [Min]	Quantity [%]	Area [µV.Sec]	#baseline
1	ethanol	0.98	0.00	29995.9	0
2	UNKNOWN	1.61	0.00	465474.5	1
Total			0.00	495470.4	

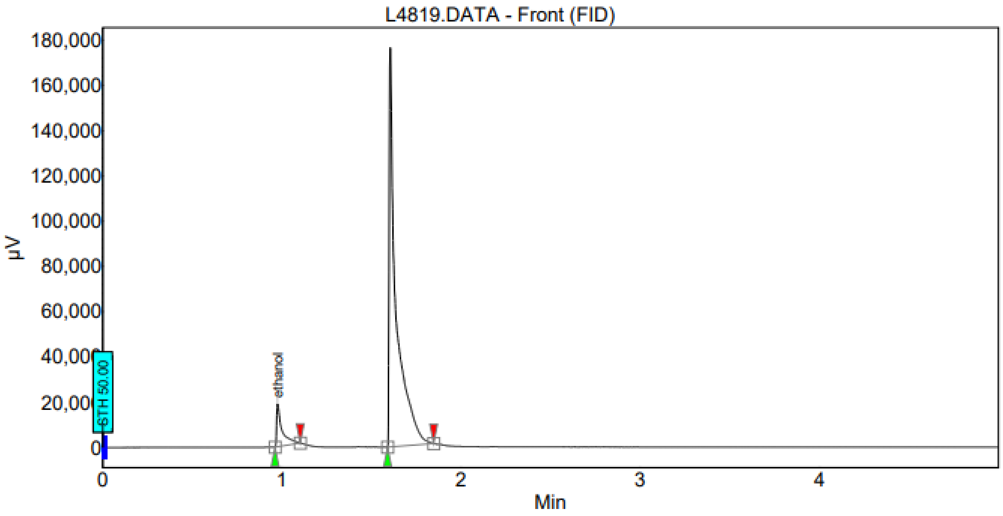

Peak results :

Index	Name	Time [Min]	Quantity [%]	Area [µV.Sec]	#baseline
1	ethanol	0.98	0.00	40153.3	0
2	UNKNOWN	1.60	0.00	470254.5	1
Total			0.00	510407.8	

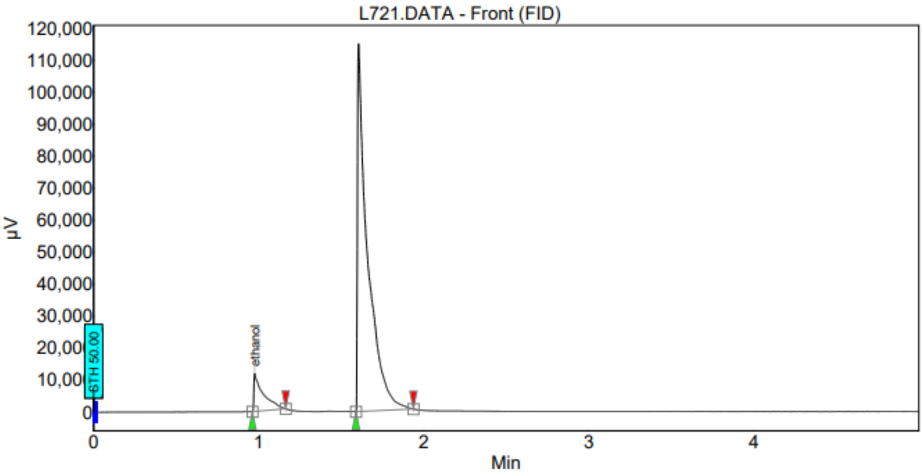

Peak results :

Index	Name	Time [Min]	Quantity [%]	Area [µV.Sec]	#baseline
1	ethanol	0.98	0.00	46721.1	0
2	UNKNOWN	1.61	0.00	483837.5	1
Total			0.00	530558.6	

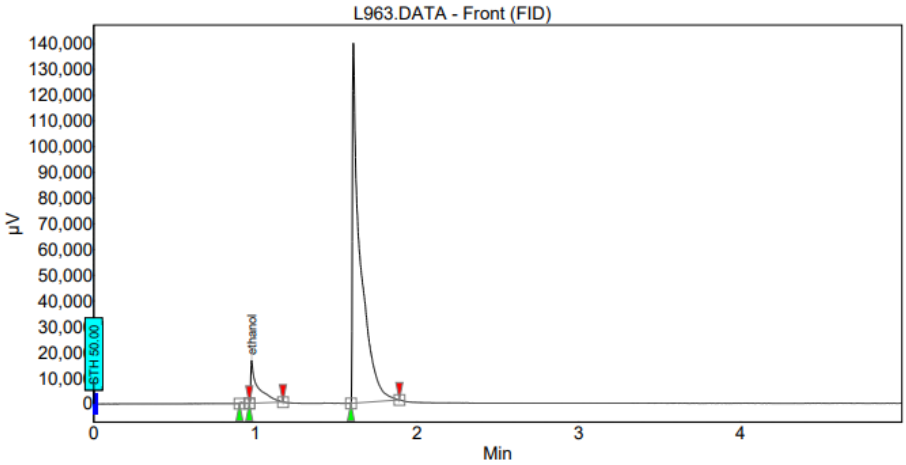

Peak results :

Index	Name	Time [Min]	Quantity [%]	Area [µV.Sec]	#baseline
1	UNKNOWN	0.91	0.00	553.6	0
2	ethanol	0.98	0.00	50246.9	1
3	UNKNOWN	1.61	0.00	469533.2	2
Total			0.00	520333.7	
